# Parkinson's disease detection using spectrogram-based multi-model feature fusion networks

**DOI:** 10.3389/fneur.2025.1706317

**Published:** 2025-11-05

**Authors:** Wenna Chen, Rongfu Lv, Xiaowei Du, Xiangyu Chen, Hao Wang, Jincan Zhang, Ganqin Du

**Affiliations:** ^1^The First Affiliated Hospital and College of Clinical Medicine of Henan University of Science and Technology, Luoyang, China; ^2^College of Information Engineering, Henan University of Science and Technology, Luoyang, China

**Keywords:** Parkinson's detection, deep learning, feature fusion, convolutional neural networks, transfer learning

## Abstract

**Introduction:**

Parkinson's disease (PD) is a common neurodegenerative disorder. Traditional diagnostic methods, relying on clinical assessment and imaging, are often invasive, costly, and require specialized personnel, posing barriers to early detection. As approximately 90% of PD patients develop vocal impairments, vocal analysis emerges as a promising non-invasive diagnostic tool. However, individual deep learning models are often limited by overfitting and poor generalizability.

**Methods:**

This study proposes a PD classification method using spectrogram feature fusion with pre-trained convolutional neural networks (CNNs). Voice recordings were obtained from 61 PD patients and 70 healthy controls (HC) at the First Affiliated Hospital of Henan University of Science and Technology. Preprocessing the raw speech signals yielded 2,476 spectrograms. Three pre-trained models, DenseNet121, MobileNetV3-Large, and ShuffleNetV2, were used for feature extraction. The output of MobileNetV3-Large was adjusted using a 1 × 1 convolutional layer to ensure dimensional alignment before features were fused via summation.

**Results:**

Evaluation using 5-fold cross-validation demonstrated that models employing feature fusion consistently outperformed individual models across all metrics. Specifically, the fusion of MobileNetV3-Large and ShuffleNetV2 achieved the highest accuracy of 95.56% and an AUC of 0.99. Comparative experiments with existing state-of-the-art methods confirmed the competitive performance of the proposed approach.

**Discussion:**

The fusion of multi-model features more effectively captures subtle pathological signatures in PD speech, overcoming the limitations of single models. This method provides a reliable, low-cost, and non-invasive tool for auxiliary PD diagnosis, with significant potential for clinical application. The code is available at https://github.com/lvrongfu/pjs.

## 1 Introduction

Parkinson's disease (PD) is a disorder of the central nervous system. It causes involuntary and uncontrollable body movements such as tremors, stiffness, or difficulties with balance and control. People with PD may also experience behavioral or mental changes, such as depression or memory decline. Currently, there is no cure for PD, but medications can alleviate symptoms. Regardless, it is preferable to intervene and prevent the progressive onset of PD rather than treat it in its most severe state. However, traditional diagnostic methods often rely on clinical assessments and imaging techniques, which can be invasive, costly, and require specialized medical expertise. In recent years, the emergence of artificial intelligence has opened new opportunities for diagnosis, particularly through voice analysis (1). Notably, since approximately 90% of PD patients exhibit speech difficulties or dysphonia, this can distinguish them from healthy individuals. Consequently, detection based on voice disorders has become a valuable, non-invasive tool for early screening of PD (2, 3).

PD affects approximately 10 million people worldwide, making it the second most common neurodegenerative disorder after Alzheimer's disease. The risk of developing PD increases with age, particularly after the age of 65 (4). Early symptoms such as loss of smell, constipation, and sleep disturbances often precede motor symptoms. Timely intervention in the early stages is crucial for slowing or halting disease progression. However, diagnosing PD based solely on clinical symptoms remains challenging and complex. Given that 90% of PD patients experience speech disorders, utilizing speech data for detection has gained prominence in recent years (5, 6). Audio signals play a pivotal role in early PD diagnosis, as speech abnormalities detectable through voice signal analysis may remain imperceptible to the human ear during initial stages. Furthermore, since voice samples can be easily recorded both clinically and non-clinically, voice changes can also serve as a means to track disease progression (7). This paper investigates the application of Deep Learning (DL) techniques in the early diagnosis of PD through the analysis of vocal characteristics to distinguish between PD patients and healthy controls (HC).

Machine learning (ML) and DL have revolutionized medical data analysis and image processing, driving significant advancements in disease diagnosis, identification, and treatment planning. Furthermore, ML enables algorithms to learn from data. As a subfield of ML, DL uses multi-layered neural networks to excel at simulating complex patterns (8). Within medical image analysis, DL approaches demonstrate exceptional efficiency in tasks related to image classification, segmentation, and anomaly detection. A key advantage of DL models lies in their ability to extract relevant features and biomarkers from diverse data sources. In voice recordings, variations in speech patterns—such as changes in pitch, rhythm, and clarity—serve as valuable biomarkers for detecting PD (9). DL, particularly convolutional neural networks (CNNs), has been extensively applied across various computer-aided diagnostic methods for medical imaging. This is because CNNs can achieve or even surpass human performance in general object recognition. Unlike traditional ML algorithms, CNNs can automatically extract meaningful features from images. Traditional algorithms, such as support vector machines and backpropagation neural networks, typically require manual feature engineering. CNNs eliminate this step, making the process more efficient and less reliant on expert knowledge. Consequently, the development of CNNs models has significantly improved the performance of computer-aided diagnostic systems. Several classification network models have been extensively applied to medical image classification tasks, including RegNet (10), ResNet (11), ConvNext (12), Vision Transformer (13), and Swin Transformer (14).

However, relying solely on a single model often leads to overfitting to the training set and poor generalization to the test set, thereby reducing the model's performance. To address this limitation, this paper proposes using model ensemble techniques. This paper utilizes three pre-trained models, namely DenseNet-121, MobileNetV3, and ShuffleNetV2, to extract features from the spectrograms of patients with PD. Subsequently, the extracted features are fused using a summation method, followed by classification of the fused features. The main contributions of this paper are as follows:

- This study proposes a spectrogram-based deep learning framework for PD classification, leveraging time-frequency representations of speech signals to capture discriminative acoustic patterns.
- The proposed novel network model enhances feature extraction capabilities by fusing features from three baseline networks.
- In extensive experimental evaluations, the proposed method has been demonstrated to outperform current techniques.

## 2 Related work

In recent years, the application of ML in PD research has advanced significantly, thanks to collaborative efforts between biomedicine and computer science. However, achieving timely and accurate diagnosis remains a major challenge. Extensive research has focused on enhancing classification performance via ML methods, using speech samples to enable automated detection of PD-related speech disorders. Typically, sustained vowel production evaluates phonetic characteristics, while continuous speech assesses articulation and intonation features (15). Numerous algorithms have been developed, and researchers have evaluated the performance metrics of different approaches (16).

Gómez-García et al. investigated the relationship between pathological voice perception and certain voice characteristics (17). Typically, in the analysis of vocalization, acoustic features are manually extracted from recordings of sustained vowels, syllable repetitions, and the reading of words, sentences, or free monologs. Selecting the most appropriate features is crucial for obtaining precise results. For instance, Mekyska et al. introduced 36 novel speech features in their study, achieving 67.9% accuracy using SVM and random forest classifiers on PD data solely through/a/vowel subset analysis (18). The Almeida team evaluated 18 feature extraction methods and four ML classifiers for PD detection and classification by analyzing sustained vowel production and other speech tasks (16). They achieved the highest accuracy of 94.55% by analyzing sustained vowel production. Carron et al. proposed a mobile-assisted speech status analysis system for PD detection, based on a server-client architecture (19). On the server side, feature extraction and ML algorithms were designed and implemented to distinguish between PD and HC. This Android application allows patients to submit voice samples, enabling physicians to review each patient's complete record. Saleh et al. (20) proposed a PD pre-diagnosis system based on Artificial Intelligence of Things (AIoT) and speech analysis. Through the sequential forward feature selection method, 10 key features were selected from 22 speech features, and the K-nearest neighbor algorithm was used for classification. Cherradi et al. (21) proposed an embedded medical system based on AIoT, which predicts PD by analyzing sound signals. This system integrates multiple machine learning algorithms and achieves an accuracy rate of 95.38%.

The aforementioned studies all employ traditional ML techniques, characterized by the need for manual feature selection. Consequently, their results are highly dependent on the quality of feature selection. To overcome this limitation, new approaches have emerged. Recent studies explored the use of spectrograms as robust inputs for deep learning models in PD detection. Spectrograms provide a time-frequency representation that preserves both temporal dynamics and spectral details, which are crucial for capturing subtle vocal impairments. For instance, Iyer et al. (7) proposed a deep learning method based on spectrograms to identify PD. The researchers used a pre-trained Inception V3 to analyze the spectrograms of continuous vowels “/a/” produced by patients and healthy individuals, achieving high-precision classification. The Berus team employed multiple neural networks to identify vocal characteristics in PD patients (22), achieving 86.47% accuracy by analyzing various speech tasks constrained by linguistic skills and experimental settings. The Rios-Urrego team utilized a CNNs-based transfer learning approach to detect PD through Mel-scale spectrogram analysis of speech data, achieving 82% accuracy (23). Hireš et al. employed an ensemble model composed of multiple CNNs to detect PD lesions in spectrogram images of vowel recordings obtained under controlled conditions (15). Optimal performance was achieved on sustained/a/vowels, yielding an AUC value of 0.89. However, most spectrogram-based approaches have focused on sustained phonations, which may not fully capture the articulatory variability present in connected speech. In contrast, our work utilizes connected speech spectrograms, which encompass a wider range of phonetic and prosodic variations, thereby offering a more naturalistic and challenging scenario for robust PD classification.

Although deep learning methods avoid the need for manual feature engineering, current research often relies on a single model architecture. This limits their ability to comprehensively capture the multi-scale and subtle pathological patterns in PD speech. Therefore, in this paper, to address the limitations of relying solely on a single model, a feature fusion technique is proposed. Three pre-trained network models are used for feature fusion, and the fused features are then classified. Experimental results show that this network achieves the expected performance in the PD classification task.

## 3 Methodology

This paper employs three pre-trained models: MobileNetV3-large, DenseNet121, and ShuffleNetV2. The outputs of these models are adjusted to ensure consistent data size, followed by feature fusion from the extracted features. Feature classification was subsequently performed. To achieve consistent output across all models' feature extraction modules, MobileNet3's feature extraction module was aligned with DenseNet121 and ShuffleNetV2 by incorporating 1 × 1 convolutional layers.

### 3.1 Dataset and pre-processing

The dataset used in this study originates from a private collection gathered by the First Affiliated Hospital of Henan University of Science and Technology. Table 1 provides demographics of the participants. All PD patients were diagnosed in accordance with the Movement Disorder Society (MDS) Clinical Diagnostic Criteria for PD (24), excluding those with comorbid neurological disorders or severe hearing/speech impairments. Participants read a passage aloud, with their voices captured to form the speech dataset. The passage reads: “On a hot summer day, we sat together under a tree by the river, enjoying the cool breeze blowing through…” Each volunteer's reading session lasted approximately 1 min and 30 s. All recordings were captured in stereo mode and saved in WAV format. In the continuous speech task, the natural pauses and silences that occur when the subjects read the paragraphs are inherent components of the speech signal. In this study, these parts were not actively removed but were retained as part of the original speech signal and used for subsequent spectrogram generation. Specifically, during the speech segmentation process, we divided the speech based on sentence pauses and paragraph endings, ensuring that each speech segment contains complete speech units and their natural pauses. Although the silent segments appear as low-frequency energy regions in the spectrogram, they, together with the speech segments, constitute the overall expression of the time-frequency characteristics. The segmentation strategy was inspired by the approach of Almeida et al. (16) in sustained vowel analysis, using sentence pauses and paragraph endings as segmentation points to preserve the naturalness and representativeness of speech segments.

**Table 1:** Demographics of participants considered in this study.

**Characteristics**	**HC (*n* = 70)**	**PD (*n* = 61)**
Sex (male/female)	42/28	38/23
Age	60 ± 10	62 ± 9

**Figure 1:**
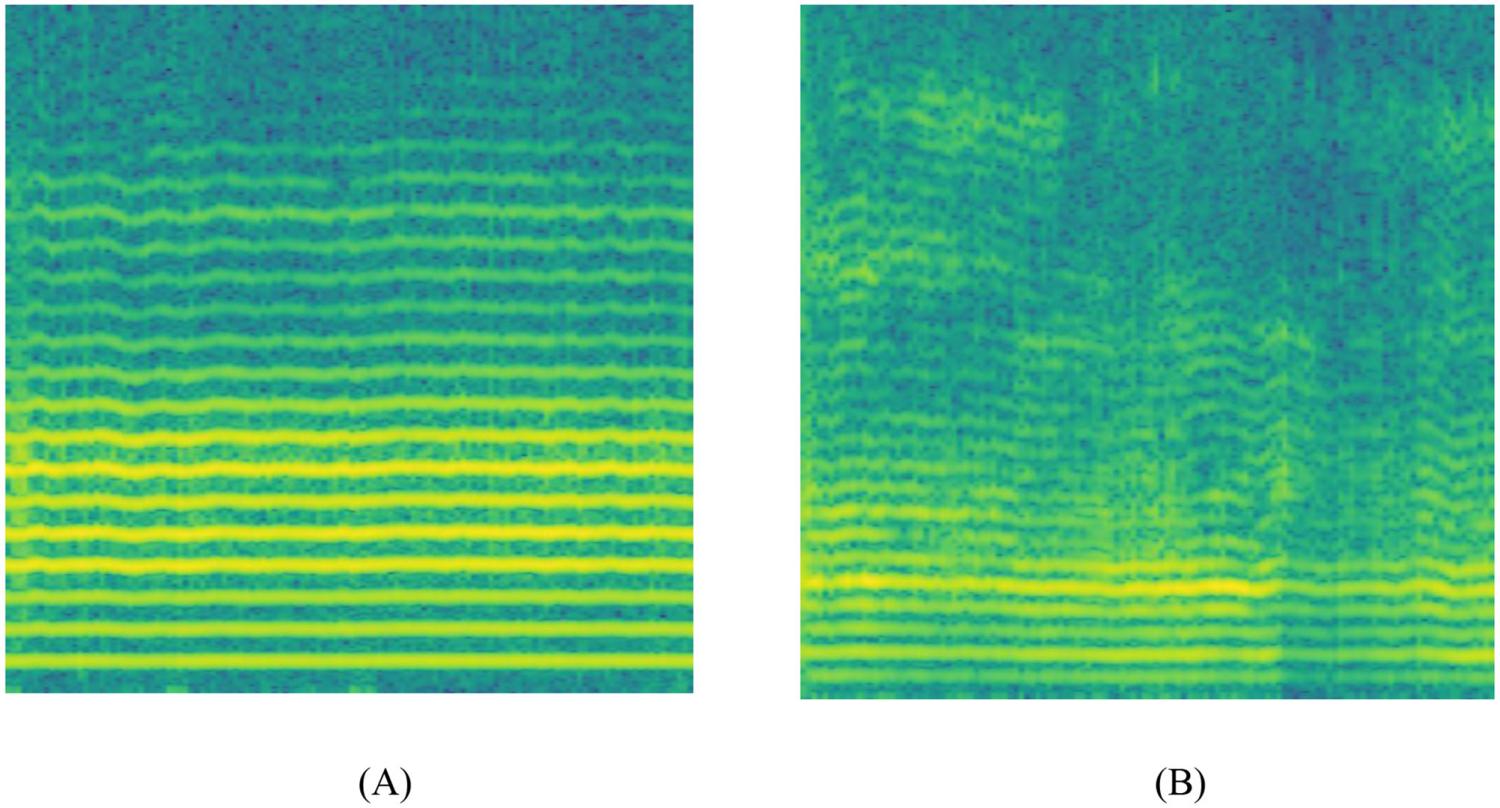
Spectrograms derived from raw audio signals. **(A)** Healthy control group, **(B)** Parkinson's disease patients.

A spectrogram is a two-dimensional heatmap depicting the frequency components of a signal over time. In speech processing, the Short-Time Fourier Transform (STFT) is commonly applied to time-domain audio signals to capture the temporal distribution of spectral characteristics. In this paper, speech segments from the dataset underwent preprocessing. An acoustic feature extraction method based on the STFT is employed to convert the raw speech signal into a spectrogram representing its time-frequency characteristics, as shown in Figure 1. Spectrograms of HC subjects (Figure 1A) exhibit clear, regular harmonic stripes, indicating stable vocal fold vibration and consistent pitch control. Their formant structures (dark horizontal bands) are equally distinct and continuous, reflecting normal phonetic precision and breath support during sustained, continuous speech. In contrast, the spectrogram of PD patients (Figure 1B) exhibits reduced regularity in harmonic structure, accompanied by increased spectral noise and formant blurring. These features indicate irregular perturbations in fundamental frequency and amplitude due to impaired neuromuscular control in PD patients; Incomplete glottic closure increases non-periodic noise in the high-frequency range, while imprecise articulation blurs the transition zones between formants. These visual patterns align with PD pathophysiological mechanisms, positioning the spectrogram as a non-invasive surrogate marker for assessing speech function deterioration. Consequently, the proposed deep learning model can learn these discriminative time-frequency features to enable automated PD detection.

Let the discrete-time audio signal be *x[n]*, with each input file resampled to 8 *kHz*. Divide *x[n]* into overlapping frames of duration *T*_*w*_ and stride *T*_*s*_. Specifically, each frame contains a fixed number of samples *N*_*w*_, and frames are separated by a sliding stride *N*_*s*_, as shown in the following formula:


(1) $$\begin{array}{l} N_{w}=T_{w}\times f_{s} \end{array}$$

(2) $$\begin{array}{l} N_{S}=T_{S}\times f_{s} \end{array}$$

Where *f*_*s*_ = *8,000 Hz, T*_*w*_ = *2.0 s, T*_*s*_ = *1.0 s*. This setting aims to balance the temporal resolution and the integrity of frequency information. The fixed frame length helps the model capture speech features on a consistent time scale, while the STFT retains sufficient details in the frequency dimension to reflect the acoustic abnormalities commonly seen in PD patients, such as minor pitch perturbations and amplitude disturbances. The formula for calculating the total number of frames in each file is:


(3) $$\begin{array}{l} N_{frames}=\max\left(1,\left\lfloor \frac{L-N_{s}}{N_{s}}\right\rfloor +1\right) \end{array}$$

Here, *L* denotes the total length of the waveform signal. If the frame extends beyond the signal's end, zero padding is applied to maintain a consistent length. For each frame *x*_*i*_*[n] (I* = 1,…, *N*_*frames*_*)*, the STFT is computed using a Hamming window of length *N*_ℓ_=*256* (i.e., *32 ms*) and a shift length *N*_*h*_ = *128* (50% overlap), with *N*_*FFT*_ = *1,024* Fourier transform points. The STFT parameter settings were aligned with the recommendations of Moro-Velázquez et al. (25) for PD speech analysis to ensure discriminative time-frequency representations. For the i-th audio segment, the Fourier coefficient (complex number) corresponding to the k-th frequency point in the m-th frame is given by the following formula:


(4) $$\begin{array}{l} X_{i}\left(k,m\right)=\overset{N_{h}-1}{\underset{n=0}{\sum }}x_{i}\left[n+mN_{h}\right]w\left[n\right]e^{-j2\pi kn/N_{FFT}} \end{array}$$

Here, *w[n]* represents the Hamming window, where *k* = *0, 1,…, N*_*FFT*_*-1*. n denotes the index of the current sample point within the window, with a range of *0*<*n*<*N*_ℓ_. The resulting amplitude spectrum matrix is:


(5) $$\begin{array}{l} S_{i}\left(k,m\right)=|X_{i}\left(k,m\right)| \end{array}$$

To enhance the dynamic range and for file normalization, each spectrogram is converted to the decibel (dB) scale:


(6) $$\begin{array}{l} S_{i,dB}\left(k,m\right)=10\log_{10}\left(\frac{S_{i}\left(k,m\right)}{\underset{k,m}{\max}S_{i}\left(k,m\right)+\varepsilon }\right) \end{array}$$

Here, ε = *10*^−10^ is used to avoid numerical instability.

Each segment's *S*_*i, dB*_ data is rendered as a color image and saved in JPEG format. This process generates multiple spectrograms per recording, containing both time-domain and frequency-domain information for subsequent neural network model training. Through this procedure, a total of 2,476 spectrograms were obtained: 1,070 from PD and 1,406 from HC. The images have dimensions of 224 × 224 pixels. Subsequently, image intensity values were normalized to the range [0,1]. Finally, the data was divided into training and test set in an 8:2 ratio. A stratified sampling strategy was employed to ensure that the proportion of PD patients to HC in both the training and test set matched that of the original dataset.

It is worth noting that the speech material used in this study consists of read passages rather than isolated sustained vowels. Although read passages contain diverse phonemes and syllable structures that may introduce variability inherent to speech content, the purpose of employing a multi-frame sampling strategy was to capture patients' overall articulation characteristics within continuous speech, rather than relying on the acoustic properties of specific phonemes. This approach offers the advantage of reflecting the patient's articulatory capabilities in a more natural and representative speech task, encompassing aspects such as pitch control, speech fluency, and syllable transitions—all of which may be affected by PD. Furthermore, despite inherent spectral differences among phonemes, deep learning models possess the ability to learn high-level abstract features from diverse inputs, enabling them to distinguish pathological from non-pathological speech patterns rather than relying solely on the spectral characteristics of specific phonemes. Existing research also supports the use of continuous speech or read passages for PD detection. For instance, Almeida et al. (16) employed both sustained vowels and continuous speech tasks in their study, noting that continuous speech provides richer articulatory dynamics information in certain scenarios. Similarly, Vásquez-Correa et al. (23) incorporated multiple speech tasks, including reading aloud, into their multimodal PD detection system, demonstrating the effectiveness of continuous speech in PD identification. Therefore, while speech content diversity may introduce additional variability, this variability does not significantly impair the model's ability to recognize PD-related speech features when addressed through appropriate model design and training strategies.

### 3.2 The architecture of the proposed method

Transfer learning is a ML technique that leverages knowledge accumulated while solving one problem to train a model for another task or domain. This transfer learning approach utilizes pre-trained network knowledge acquired from vast amounts of visual data, offering significant advantages in time savings and improved accuracy compared to training models from scratch (26). Transfer learning is particularly advantageous when dealing with limited data, as pre-trained models bring robust feature representations learned from large-scale datasets that capture generic visual patterns such as edges, textures, and shapes (27). These representations can be effectively adapted to domain-specific tasks, even with a modest number of samples.

**Figure 2:**
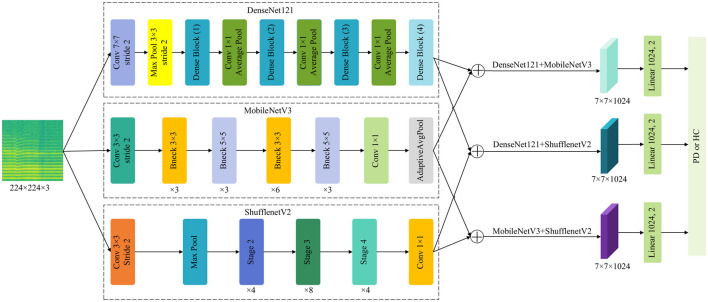
The proposed network model architecture.

CNNs have been widely applied to image-related tasks such as image recognition and image classification. The use of CNNs has effectively improved the performance of many image-related tasks (28). This study employs a CNNs based on feature fusion, thereby enhancing the network's ability to extract features. Figure 2 illustrates the framework of the proposed method. First, PD data undergoes processing, and images are adjusted. Next, feature extraction from spectrograms is performed using pre-trained models. Finally, extracted features are aggregated for feature fusion before classification. Specifically, DenseNet121, MobileNetV3, and ShuffleNetV2 serve as pre-trained network models. The output from the feature extraction layer of MobileNetV3 is reshaped to dimensions (1024, 7, 7). Feature fusion via summation was selected after empirical comparison with alternatives including concatenation, weighted averaging, and attention-based fusion. Summation preserves the spatial dimensions of feature maps while reducing computational complexity compared to concatenation. It also encourages the model to integrate complementary information from different networks without introducing additional parameters, as would be required by attention mechanisms. After extracting features from the spectrogram, the features of each model are summed up pairwise to form a fused feature representation. In this study, a pairwise model fusion approach was adopted instead of a three-model fusion approach. The main reason was to avoid a significant increase in computational burden and potential feature redundancy. The experiments demonstrated that pairwise fusion was able to fully capture complementary information and achieve excellent performance. This fused feature map is then passed to a linear classifier consisting of a fully connected layer followed by a softmax activation function, which outputs the final probability distribution over the two classes. The fusion strategy employed is thus feature-level summation. This approach allows the model to leverage complementary information from different architectures directly in the feature space, enhancing the discriminative power for PD classification.

### 3.3 Pre-trained models

In this study, pre-trained refers to CNN models that were initially trained on large-scale external datasets unrelated to PD or medical voice data. The three pre-trained models employed in this study were all pre-trained on the ImageNet dataset. ImageNet is a large-scale visual recognition dataset comprising over 14 million annotated images across 1,000 object categories, widely used for model pre-training in image classification tasks (29).

In typical CNNs, the convolutional layer extracts local features by sliding a fixed-size convolution kernel over the input tensor and performing element-wise multiplication of the kernel weights with corresponding input regions. Beyond the kernel parameters, the stride, padding, and kernel size jointly determine the spatial transformation of the feature map. The output spatial size of the convolutional layer is given by the following formula:


(7) $$\begin{array}{l} H_{out}=\frac{H_{in}+2p-k}{s}+1,W_{out}=\frac{W_{in}+2p-k}{s}+1 \end{array}$$

Here, *H*_*in*_ and *W*_*in*_ represent the height and width of the input feature map, respectively; *p* denotes the number of zero-padding pixels on each side; *k* indicates the size of the convolutional kernel; and *s* signifies the stride of the convolution. When *k* = *1, s* = *1*, and *p* = *0*, the spatial dimensions of the output feature map remain unchanged, thereby helping to preserve high-resolution details.

The selection of DenseNet121, MobileNetV3-Large, and ShuffleNetV2 is based on their complementary architectural strengths for spectrogram analysis. DenseNet121 excels in feature propagation and reuse, ideal for capturing complex spectral patterns. MobileNetV3-Large offers a superior balance between accuracy and computational efficiency via depthwise convolutions and attention mechanisms. ShuffleNetV2 is a highly efficient model designed for low-resource environments, using channel shuffling to maintain representational power. Integrating these diverse models allows our fusion method to leverage their distinct advantages, thereby more comprehensively capturing the multi-scale pathological features in PD speech.

**Figure 3:**
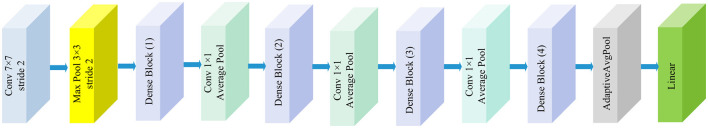
Structure of the DenseNet121 model.

DenseNet was proposed by Huang et al. (30). The core idea of DenseNet is to introduce dense connections within the network. This design significantly enhances the capabilities of feature propagation and reuse, while also effectively mitigating the vanishing gradient problem. Figure 3 illustrates the basic framework of the DenseNet121 model. In the DenseNet architecture, each layer is directly connected to all preceding layers. Specifically, layer *l* receives all feature maps from the previous *l*−*1* layers as input, and its output is also fed to all subsequent layers. The output dimension of the feature extraction layer is (1024, 7, 7). This structure can be represented as:


(8) $$\begin{array}{l} x_{l}=H_{l}\left(\left[x_{0},x_{1},\ldots ,x_{l-1}\right]\right) \end{array}$$

Here, *x*_*l*_ denotes the output of layer *l*; *H*_*l*_*(*·*)* represents the nonlinear transformation function comprising batch normalization, the ReLU activation function, and convolution; *[x*_0_*,x*_1_*,…,x*_*l*−1_*]* denotes the concatenation of feature maps.

**Figure 4:**
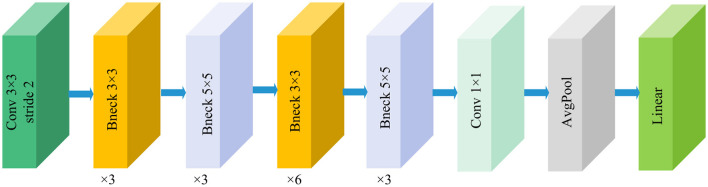
Structure of the MobileNetV3-Large model.

MobileNetV3 is a lightweight network architecture proposed by the Google team (31), representing a significant upgrade following MobileNetV1 and V2. Figure 4 illustrates the fundamental framework of the MobileNetV3 model. Its design comprehensively integrates multiple optimization strategies. These include depthwise separable convolutions, inverted residual structures, squeeze-and-excitation channel attention mechanisms, and an efficient network architecture. This architecture compresses parameters and reduces computational complexity while preserving strong feature representation capabilities. As one of the representative models in the current edge computing field, it achieves a balance between precision and efficiency. The feature output dimension from the feature extraction layer of the MobileNetV3 model is (960, 7, 7). To enable feature fusion with DenseNet121 and ShuffleNetV2, a 1 × 1 convolutional layer with 1,024 convolutional kernels is added to the base model, adjusting the feature extraction layer output dimension to (1024, 7, 7).

**Figure 5:**
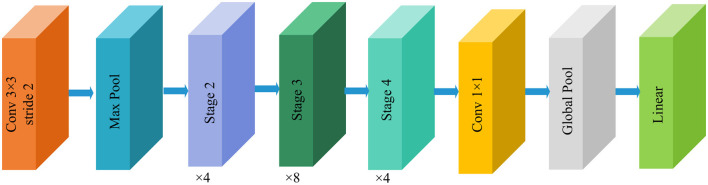
Structure of the ShuffleNetV2 model.

ShuffleNetV2 is an efficient, lightweight CNNs proposed by Megvii Technology team (32), designed to achieve faster inference speeds and lower resource consumption on mobile and embedded devices. Its core concept lies in breaking communication barriers between different groups through channel grouping and feature channel shuffling operations, thereby enhancing feature flow efficiency and network expressiveness. Structurally, the basic unit of ShuffleNetV2 comprises two branches. The outputs from both branches undergo channel concatenation and are combined with channel shuffle operations to facilitate information exchange, achieving parameter efficiency and computational friendliness. Figure 5 illustrates the basic framework of the ShuffleNetV2 model. This structure is stacked repeatedly to build the complete network, offering excellent scalability. The output dimension of the feature extraction layer is (1024, 7, 7).

## 4 Results

The experimental environment in this study utilized a Windows 10 operating system with 64GB of random access memory, Python 3.10.15, and PyTorch 2.1.0. Employing a cosine annealing scheduling strategy. Hyperparameters were primarily selected based on established defaults for the Adamw optimizer and adjusted through manual trial and error guided by performance on a held-out test set. Detailed information regarding the hardware configuration and model parameters is provided in Table 2.

**Table 2:** Hardware configuration and model parameters.

**Types**	**Configuration**	**Types**	**Value**
GPU	RTX 4070	Init-lr	5e-4
CPU	I5-13400F	Weight_decay	5e-2
CUDA	12.0	Epoch	100
Pytorch	2.1.0	optimizer	Adamw
Regularization	Dropout	Batch size	16

### 4.1 Evaluation metric

To comprehensively evaluate the model's effectiveness, this paper employs evaluation metrics including accuracy, precision, recall, F1-score, and specificity. The expressions for these evaluation metrics are shown in Equations 9–13.


(9) $$\begin{array}{l} \text{Accuracy}=\frac{TP+TN}{TP+TN+FP+FN} \end{array}$$

(10) $$\begin{array}{l} \text{Precision}=\frac{TP}{TP+FP} \end{array}$$

(11) $$\begin{array}{l} \text{Recall}=\frac{TP}{TP+FN} \end{array}$$

(12) $$\begin{array}{l} F1-score=2\times \frac{Precision\times Recall}{Precision+Recall} \end{array}$$

(13) $$\begin{array}{l} \text{Specificity}=\frac{TN}{TN+FP} \end{array}$$

Among these, TP (True Positives) represents the number of correctly predicted positives; TN (True Negatives) represents the number of correctly predicted negatives; FP (False Positives) represents the number of incorrectly predicted positives; and FN (False Negatives) represents the number of incorrectly predicted negatives.

### 4.2 Experimental results

This section presents the classification results obtained by the proposed method through 5-fold cross-validation, and conducts a comparative analysis between the use and non-use of the feature fusion method.

#### 4.2.1 The classification results of a single model

**Figure 6:**
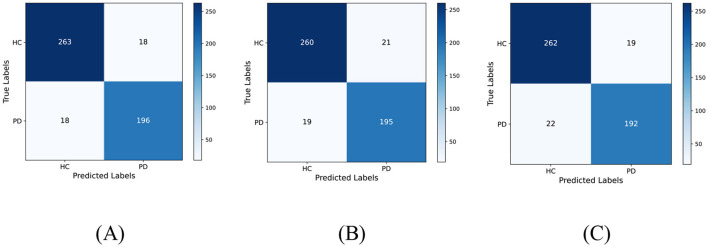
Confusion matrix of predicted results for a single model on the test set. **(A)** DenseNet121 **(B)** MobileNetV3, and **(C)** ShuffleNetV2.

To more intuitively reflect the overall classification performance of the models, the confusion matrices of the three pre-trained models on the dataset are plotted as shown in Figure 6. Additionally, Table 3 lists the specific values of accuracy, precision, recall, F1-score, and specificity calculated using Formulas 9–13. Table 3 indicates that DenseNet121 demonstrates the best PD classification performance, achieving an accuracy of 92.73%.

**Table 3:** Indicators for the classification of a single model.

**Model**	**Type**	**Precision (%)**	**Recall (%)**	**F1-score (%)**	**Specificity (%)**	**Accuracy (%)**
DenseNet121	HC	93.59	93.59	93.59	91.59	
	PD	91.59	91.59	91.59	93.59	
	Average	92.59 (±0.02)	92.59 (±0.02)	92.59 (±0.04)	92.59 (±0.01)	92.73 (±0.03)
MobileNetV3	HC	92.53	93.19	92.86	90.28	
	PD	91.12	90.28	90.70	93.19	
	Average	91.83 (±0.01)	91.74 (±0.02)	91.78 (±0.03)	91.74 (±0.02)	91.92 (±0.02)
ShuffleNetV2	HC	93.24	92.25	92.74	91.00	
	PD	89.72	91.00	90.35	92.25	
	Average	91.48 (±0.02)	91.63 (±0.04)	91.55 (±0.03)	91.63 (±0.01)	91.72 (±0.02)

#### 4.2.2 The classification results after feature fusion

**Figure 7:**
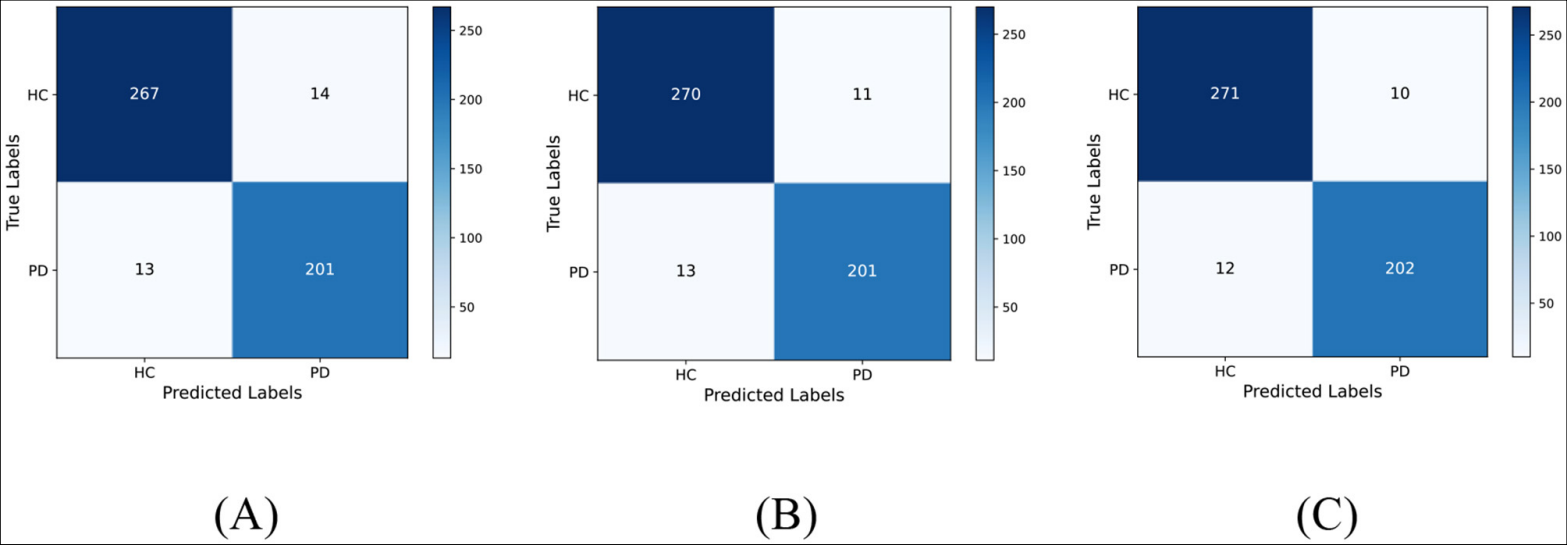
Classification results of PD on the test set. **(A)** DenseNet121+ShuffleNetV2 **(B)** DenseNet121+MobileNetV3, and **(C)** MobileNetV3+ShuffleNetV2.

Figure 7 displays the confusion matrix for PD classification results achieved through feature fusion. The diagonal region representing correctly classified cases exhibits distinct dark clustering characteristics, with numerical values highly concentrated along the diagonal. Additionally, Table 4 provides detailed metric values evaluated on the dataset. It can be observed that MobileNetV3+ShuffleNetV2 achieved the best classification results on the dataset with an accuracy of 95.56% and an Area Under Curve of 99%. This validates the effectiveness and reliability of this model for PD classification tasks.

**Table 4:** The classification results of feature fusion methods.

**Model**	**Type**	**Precision (%)**	**Recall (%)**	**F1-score (%)**	**Specificity (%)**	**Accuracy (%)**	**AUC (%)**
DenseNet121+ShuffleNetV2	HC	95.02	95.36	95.19	93.49		98
	PD	93.93	93.49	93.71	95.36		
	Average	94.48 (±0.01)	94.43 (±0.02)	94.45 (±0.04)	94.43 (±0.02)	94.55 (±0.03)	
DenseNet121+MobileNetV3	HC	96.09	95.41	95.74	94.81		99
	PD	93.93	94.81	94.37	95.41		
	Average	95.01 (±0.02)	95.11 (±0.03)	95.06 (±0.05)	95.11 (±0.02)	95.15 (±0.04)	
MobileNetV3+ShuffleNetV2	HC	96.44	95.76	96.10	95.28		99
	PD	94.39	95.28	94.84	95.76		
	Average	95.42 (±0.01)	95.52 (±0.02)	95.47 (±0.03)	95.52 (±0.02)	95.56 (±0.02)	

**Figure 8:**
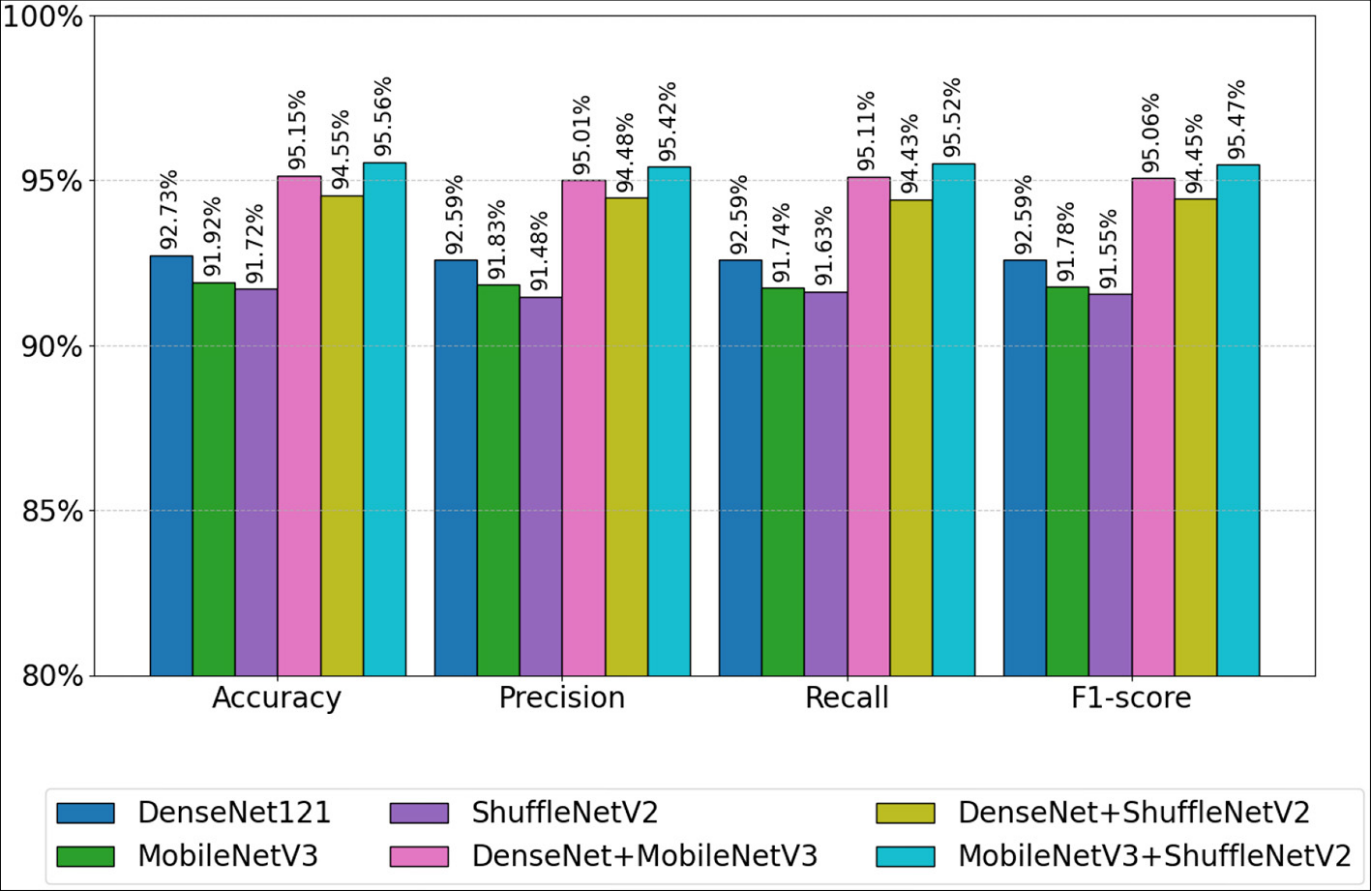
Visualization of PD classification metrics.

In order to more intuitively demonstrate the advantages of the fusion model, Figure 8 shows the average evaluation metrics for PD classification across each model. It can be observed that the combination of MobileNetV3 and ShuffleNetV2 (MobileNetV3+ShuffleNetV2) achieves the best classification accuracy, precision, recall, and F1-score, at 95.56%, 95.42%, 95.52%, and 95.47%, respectively. Additionally, among individual models, DenseNet121 demonstrated the best classification results for PD. All performance metrics of the feature-fused hybrid models outperformed the respective metrics of the individual models. The experimental results validate the effectiveness of combining features from different models through feature fusion. This provides a more reliable approach for PD classification than relying on a single model.

#### 4.2.3 Robustness validation

Based on the foregoing, it is evident that the feature fusion model yields superior classification results across the private dataset. This section aims to evaluate the model's robustness by validating it on the publicly available PD speech dataset from Figshare (33). Using the same data processing methodology, a total of 1,034 spectrograms were obtained. Among these, 536 were from PD patients and 498 from HC. Results from 5-fold cross-validation are presented in Table 5. The findings indicate that high accuracy is maintained even on the public dataset, demonstrating the model's robustness.

**Table 5:** Classification results of feature fusion methods on public datasets.

**Model**	**Precision (%)**	**Recall (%)**	**F1-score (%)**	**Accuracy (%)**	**AUC (%)**
DenseNet121+ShuffleNetV2	90.11 (±0.01)	91.74 (±0.02)	90.86 (±0.04)	92.43 (±0.04)	94.78
DenseNet121+MobileNetV3	91.45 (±0.02)	89.60 (±0.01)	90.46 (±0.03)	92.43 (±0.03)	93.63
MobileNetV3+ShuffleNetV2	90.46 (±0.02)	89.75 (±0.03)	90.09 (±0.02)	92.03 (±0.02)	93.62

### 4.3 Comparison with other state-of-the-art methods

To further validate the effectiveness of the proposed method, this study conducted comprehensive comparative experiments between the proposed network and a series of state-of-the-art models. In order to conduct fair and direct comparisons under the same conditions, we implemented the core algorithms and architectural frameworks from each literature, and applied them to our own dataset for training and evaluation. The experimental results are shown in Table 6. The results demonstrate that the proposed model exhibits outstanding performance, achieving an accuracy of 95.56%, precision of 95.42%, recall of 95.52%, F1-score of 95.47%, and specificity of 95.52%, outperforming all other models. The comparative results presented in Table 5 indicate that our research achieves competitive classification performance compared to the state-of-the-art methods in the current literature.

**Table 6:** Comparison with other state-of-the-art models.

**Model**	**Accuracy (%)**	**Precision (%)**	**Recall (%)**	**F1-score (%)**	**Specificity (%)**
Han et al. (36)	88.69	88.76	88.39	88.54	88.39
Radosavovic et al. (37)	88.08	87.28	88.46	87.69	88.46
Sunkara et al. (38)	82.63	82.24	82.28	82.34	82.28
Yu et al. (39)	93.33	93.24	93.19	93.22	93.19
Woo et al. (40)	89.09	88.72	89.01	88.85	89.01
Ma et al. (41)	90.51	90.52	90.24	90.37	90.24
Huo et al. (42)	91.72	91.54	91.43	91.56	91.43
Huo et al. (43)	80.81	81.26	80.70	80.70	80.70
Yu et al. (44)	91.11	91.11	90.87	90.98	90.87
Ding et al. (45)	89.90	90.10	89.61	89.79	89.61
Li et al. (46)	90.51	90.36	90.32	90.33	90.32
Proposed	95.56	95.42	95.52	95.47	95.52

## 5 Discussion

This study investigates the effectiveness of pre-trained CNNs and feature fusion strategies in PD classification based on spectrograms, yielding several important findings worthy of further exploration.

The superior performance of the DenseNet121 model, which achieved a peak accuracy of 92.73%, is attributable to its densely connected architecture. As detailed in Section 3.3.1, this design enhances feature propagation and reuse, effectively mitigates the vanishing gradient problem, and promotes robust feature integration. These properties likely explain its efficacy in capturing the subtle acoustic patterns indicative of PD voice impairment. In contrast, the lightweight architectures of MobileNetV3-Large and ShuffleNetV2—optimized for computational efficiency on edge devices—achieved slightly lower accuracies of 91.92% and 91.72%, respectively. This performance discrepancy illustrates a fundamental trade-off between model efficiency and representational capacity. While MobileNet3's squeeze-and-excite modules and ShuffleNet2's channel shuffling operation prioritize inference speed, DenseNet11's structure favors comprehensive feature extraction.

The experimental results demonstrate an improvement in the performance of the feature fusion model. This enhancement indicates that integrating features from models based on distinct design philosophies can strengthen discriminative capabilities. The fusion model consistently outperforms results from individual models, highlighting the limitations of relying on features extracted from a single model architecture for PD classification. Pathological voice characteristics in PD may manifest across a range of spectral bands (e.g., low-frequency harmonics, high-frequency noise components) and temporal segments (e.g., vowel pronunciation, transitional speech segments), which are not equally captured by all models.

Despite the aforementioned strengths of this study, limitations remain. The dataset originates from a single institution, comprising 61 PD patients and 70 HC, with speech recordings confined to a single text. This may limit the study's generalizability, as speech patterns can vary across dialects, age groups, and task complexity. Future research should focus on expanding datasets to incorporate diverse linguistic materials and populations, exploring cross-lingual transfer learning, and integrating additional modalities (such as gait or handwriting.) to achieve multimodal PD diagnosis (34, 35).

In conclusion, this study demonstrates that the feature fusion of pre-trained CNNs can improve the performance of PD classification based on spectrograms, providing a promising approach for auxiliary diagnosis. The research results emphasize the importance of multi-model integration in capturing complex pathological biomarkers and also lay the foundation for the development of deployable PD screening tools.

## 6 Conclusion

This study aims to overcome the limitations of traditional PD diagnostic methods, including their invasiveness, high cost, and dependence on specialist expertise. Additionally, it addresses the drawbacks of single DL models in voice-based diagnosis to pursue a non-invasive and reliable auxiliary diagnostic solution. To this end, this study utilized the dataset comprising 61 PD patients and 70 HC. The raw speech signals were preprocessed using STFT to generate 2,476 spectrograms. Three pre-trained CNNs—DenseNet121, MobileNetV3-Large, and ShuffleNetV2—were selected for feature extraction. To ensure consistent output dimensions with the other two models, the output dimension of MobileNetV3-Large was adjusted to (1024, 7, 7) via a 1 × 1 convolutional layer, followed by feature fusion through summation. Experimental results demonstrated that all feature fusion models outperformed single models. Among the single models, DenseNet121 achieved the highest accuracy at 92.73%. The fusion model combining MobileNetV3-Large and ShuffleNetV2 achieved optimal classification performance with an accuracy of 95.56% and an AUC value of 0.99, outperforming existing state-of-the-art methods reported in the literature. This result confirms that integrating CNNs models with distinct architectural strengths enables more comprehensive capture of subtle pathological speech patterns in PD patients, thereby enhancing diagnostic accuracy. Although this study contributes to providing a non-invasive PD diagnostic aid, limitations exist. For instance, speech recordings were restricted to a single language, potentially affecting the study's generalizability. Future work will focus on expanding datasets to include diverse populations and linguistic materials. Cross-lingual transfer learning and integrating multimodal data will be explored to further improve the model's diagnostic performance and clinical applicability.

## Data Availability

The datasets presented in this article are not readily available because the data that has been used is confidential. Requests to access the datasets should be directed to Wenna Chen: chenwenna0408@163.com.
